# Multidirectional Instability: Natural History and Evaluation

**DOI:** 10.2174/1874325001711010861

**Published:** 2017-08-31

**Authors:** Miguel García Navlet, Cristina Victoria Asenjo-Gismero

**Affiliations:** 1 Shoulder and Elbow Unit, Upper extremity department at ASEPEYO Hospital Coslada, Madrid, Spain; 2 Orthopedic Resident at ASEPEYO Hospital Coslada, Madrid, Spain

**Keywords:** Multidirectional instability, Laxity, Hyperlaxity, Shoulder instability, Scapular dyskinesia

## Abstract

**Background:**

Multidirectional instability (MDI) represents a great challenge to the orthopedic surgeon. When treating these patients we must be aware that instability refers to a symptomatic situation, thus multidirectional instability is defined as symptomatic involuntary instability in two or more directions, and should be clearly differentiated from asymptomatic hyperlaxity.

It may be associated with hyperlaxity, either congenital or acquired following repetitive stress, but also may be present without hyperlaxity, which is rare.

**Methods:**

We searched in the online data bases and reviewed the relevant published literature available.

**Results:**

Many differences can be seen in the current literature when identifying these patients, unclear definitions and criteria to be included in this patient group are common.

**Conclusion:**

Understanding the complex shoulder biomechanics as well as being aware of the typical clinical features and the key examination signs, which we review in this article, is of paramount importance in order to identify and classify these patients, allowing the best treatment option to be offered to each patient.

## 1 INTRODUCTION

Identifying patients with multidirectional instability (MDI) is of paramount importance when treating unstable shoulders. Since they represent a great challenge and regular treatment techniques and strategies for simple anterior instability will not solve the patient´s problem if we fail to identify this condition.

Joint hypermobility was first described by Hippocrates in Scithyan warriors from central Asia. But it is not until Kirk´s paper in 1965, describing the hypermobility syndrome, that modern scientific literature starts paying attention to symptoms related with generalized hypermobility [1].

Neer and Foster coined the term “Multidirectional Instability” in 1980 [2]; although previous reports highlighted the importance of recognizing these patients, their landmark article demonstrated the lack of consensus, to that date, on the management of this condition. Although great interest in literature has led to an increase about the knowledge on how the shoulder works and how dysfunction is established, little has changed ever since, as far as a consensus on dealing with this complex situation.

Nevertheless, the exact relation of generalized joint laxity and shoulder instability continues to be debated.

*“In 1992, Harryman et al*. [3]*; used a rigorous methodology to demonstrate convincingly that normal individuals can have a great deal of capsular laxity without any symptoms whatsoever *[4].”

Instability refers to a symptomatic situation, thus multidirectional instability is defined as symptomatic involuntary instability in two or more directions which differs from hyperlaxity that is characterized by increased length and elasticity of normal joint restraints, resulting in a greater degree of translation of the articular surfaces [5], but still, physiological and asymptomatic.

Hyperlaxity can be either congenital or acquired. Congenital is usually, but not necessarily, related to connective tissue disorders such as Ehlers-Danlos syndrome, Marfan syndrome, benign hypermobility syndrome and osteogenesis imperfecta [6]. Acquired is more typically related to sports in which athletes are exposed to repetitive microtrauma and overuse, as in gymnastics, swimming and throwing.

Many ways have been described to identify hyperlaxity. Although Beighton´s [5] criteria (Table **1**) is probably the most widely used scoring system, we find the Bulbena´s Hospital del Mar Score (Table **2**) [7] to be more complete. It brings together all previous relevant scores, including, what´s more interesting to us, the “shoulder external rotation higher than 85º” criteria, which Chahal [8] proved to be a predisposing factor for anterior shoulder dislocation and it also includes the Beighton´s criteria which have also been proven to confirm the association between hyperlaxity and shoulder dislocation [9].

Since the classical Matsen´s TUBS and AMBRI classification did not include hyperlaxity criteria within the groups, Gerber and Nyffeler in 2002 [10], classified dynamic instability describing two groups of multidirectional instability, whether it´s associated with hyperlaxity or not (Table **3**).

In the setting of a suspected multidirectional instability, we should be able to identify or be aware of 5 different clinical settings, excluding those with rheumatic disorders related to joint hyperlaxity:

- People with generalized joint hyperlaxity, with no complaints.
- Patients with a benign hypermobility syndrome, in which systemic rheumatic diseases can be excluded, but present with pain in multiple joints, and may be associated to joints dislocation or subluxation, as well as extrarticular manifestations.
- Patients with shoulder dislocation in one direction with joint hypermobility or hyperlaxity. That is not multidirectional instability.
- Patients with shoulder dislocation in two or more directions, thus multidirectional instability, either with or without joint hypermobility or hyperlaxity.
- Voluntary Instability with hyperlaxity. Gerber and Nyffeler´s type C, that is divided in three groups where C1 are people who don´t suffer for this, they are just surprised they can do it, meaning an over the average control of the shoulder. C2 are the voluntary dislocators with symptoms, they start with an involuntary dynamic instability and learn how to subluxate and reduce it afterwards, this group should be considered as the dynamic B6 group (Uni or Multidirectional with voluntary reduction) and be treated correspondingly. And the C3, very important to identify since they dislocate to gain attention and it´s an expression of psychiatric illness [10].

Focusing on multidirectional instability, we can consider three types, the first one is related to generalized hyperlaxity, the second one is related to overuse, generally athletes (swimmers, gymnasts) who develop hyperlaxity in their shoulder because of repetitive stress. These two may be considered atraumatic instabilities, thus Multidirectional Instability with Hyperlaxity, and usually present with an insidious onset of subtle symptoms. There is a third type that results from multiple injuries to different areas of the shoulder, or a significant trauma, thus Multidirectional Instability without Hyperlaxity [11], this is a rare condition where symptoms are related to a traumatic onset, usually with anterior and posterior instability with no signs of inferior laxity in the affected shoulder, and no laxity signs in the contralateral shoulder [3].

According to the dislocation direction, we may differentiate three groups: Antero-inferior dislocation with posterior subluxation, postero-inferior dislocation with anterior subluxation and global dislocation [12].

### 1.1 Anatomy and Biomechanics

It is known that stability of the shoulder is provided by the static (glenoid concavity and version, labral height and glenohumeral ligaments) and the dynamic stabilizers (scapulothoracic muscles, rotator cuff, proprioceptive and neuromuscular control), in multidirectional instability both mechanisms are usually altered in order to cause symptoms in two or more directions. But interestingly, isolated changes of the dynamic stabilizers exist but no isolated changes of the static stabilizers has been found [13].

But even more important is to have in mind the concept of functional stability of the shoulder, that is, the interconnection of the stabilizing mechanisms during shoulder motion, where a coordinated activation of numerous muscles is needed in order to produce movement while maintaining joint stability by keeping the concavity-compression mechanism active throughout the range of motion [14, 15].

Stability at rest is provided by negative pressure that is created by corresponding surfaces, a watertight capsule and joint fluid. During motion, stability is granted by the balance created by muscle activation that will create a synchronous scapulo-humeral movement, allowing to achieve the more efficient position of the scapula relative to the humerus on every position of the arm during motion. And it is not until the end, at the extreme range of motion that the capsular and ligamentous restrains play a major role [16].

Along with the static stabilizers, the dynamic stabilizers are essential since only the neurally mediated muscle activation control has the capacity to adapt joint mechanics in response to the different limb configurations and loading conditions [17]. The function of both joint stabilizers is integrated by the proprioception mechanism.

Articular proprioception comprises both movement perception (kinesthesia) and joint position awareness. This kind of neuromuscular control may become dysfunctional when the nervous reflex is disrupted. An injury to articular structures containing mechanoreceptors affects proper signaling to the central nervous system [18]. In 2004, Barden *et al*. [19], found that subjects with multidirectional shoulder instability were unable to use proprioception to reposition the hand as accurately as a control group, regardless of the type of movement studied, representing a reduced capacity to use proprioception to refine and control the motor output of the upper limb. A deficiency in the capacity to generate proprioceptive feedback would provide a potential mechanism for how atypical shoulder muscle activity might occur in multidirectional instability patients.

Patulous and redundant inferior capsular structures have been widely pointed out as a hallmark of multidirectional instability, and it’s been showed in MR arthrography that an increase dimension of the postero-inferior and inferior capsule, is a constant finding in symptomatic multidirectional unstable patients [20]. However this is not enough to produce symptoms, since it´s also been found in asymptomatic shoulders and up to 23% of fetal and embryonic shoulders, pointing out that its etiology may be developmental not just traumatic [21], and most patients are not symptomatic from birth. Even more, the study of Lippit *et al*. [22] found similar glenohumeral translational measurements when comparing patients with MDI and an asymptomatic control group.

The biochemical and histologic properties of the capsule have also been studied, so far with no clear conclusions since unidirectional and multidirectional instabilities present similar characteristics, but the suspicion of an underlying connective tissue disorder still remains [23].

**Fig. (1):**
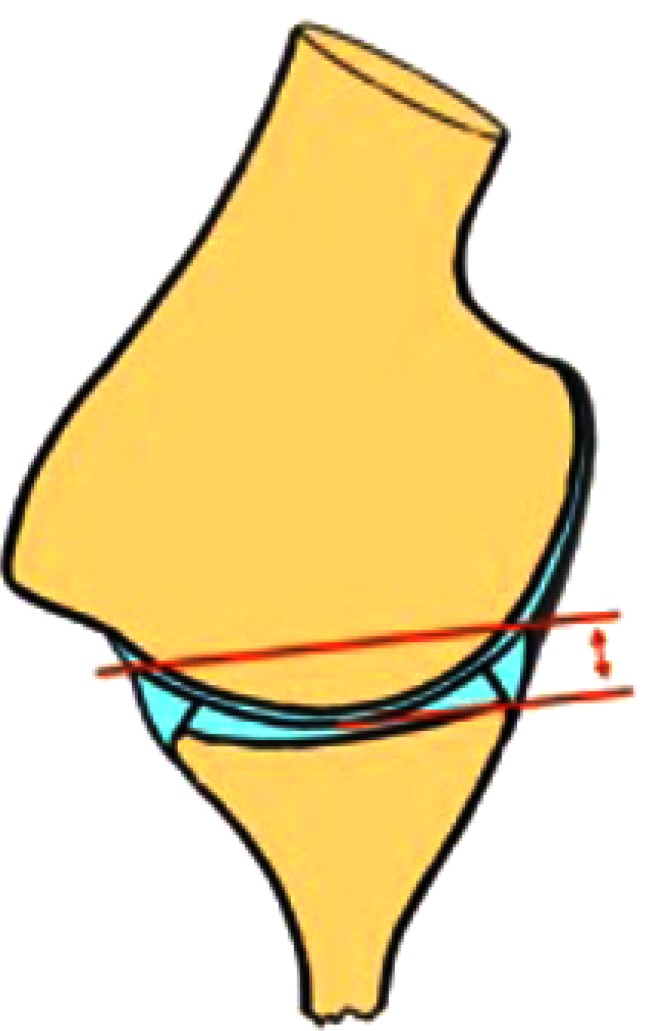
Effective Glenoid Depth. Meassured from the deepest part of the glenoid to the highest labral height.

The static effect of the glenoid concavity compression mechanism is proportional to the effective glenoid depth (Fig. **1**) [14]. Significant alterations of the static stabilizers with an increased glenoid retroversion and flattening of the chondrolabral posteroinferior portion, in the middle and inferior planes of the glenoid, are often found in these patients [24]. However, the extent of these changes varies widely and a relationship between these changes and the direction of the instability has not been found, it remains unclear if this labral retroversion is cause or consequence of the instability.

These results highlight the importance of the dynamic stabilizers as causative in atraumatic and multidirectional instability, where improper alignment of the scapula combined with the inherent instability of the glenohumeral joint may cause excessive translational movement within the glenohumeral joint and may increase the risk for additional micro or macro injury [25].

We should be aware that not only the shape of the osseous components is important, position of the glenoid has also been proved to increase posterior instability when tilted more than 15° posteriorly, and anterior instability increased when tilted 5° anteriorly [26].

Furthermore, it´s been proved that multidirectional instability displays a typical alteration in the scapular kinematics consisting in a decrease in upper rotation and increase of internal rotation, in a combined movement of protraction of the scapula, during elevation of the arm in the scapular plane throughout all phases [16]. This scapular protraction is associated with inhibition of the subscapularis, lower trapezius and serratus anterior, coupled with increased activation of pectoralis minor and latissimus dorsi [16].

So any diminution of the effective depth of the inferior part of the glenoid either by shape, static position or dynamic position due to muscle activation and scapular dyskinesis [27] may play a role in multidirectional instability, and should be considered during the diagnostic workup and when assessing possible treatment options.

Electromyographic analysis in multidirectional instability patients, show the importance of muscle control and highlights the relevance of the combined alteration of the static and dynamic stabilizers.

Regarding muscle activation patterns, the literature findings suggest that in the rotator cuff the activity levels are the same in multidirectionally unstable patients and control subjects [28]; but is the timing of the recruitment what is affected by an asynchronous pattern of activation, presenting a premature deactivation of the infraspinatus and supraspinatus muscles in subjects with multidirectional instability, particularly in the externally rotated and abducted position, experiencing a loss of dynamic stability at a critical point in the range of motion [29]. The posterior deltoid shows a delayed onset of activation, compromising anterior stability in internal rotation. The pectoralis major maintains a low level of activity during great part of the flexion/extension range of motion, failing to create different patterns of activation/deactivation for each of the separate phases as happens in normal subjects [29]. The latissimus dorsi appears to be the most dominant muscle involved in multidirectional instability, due to overactivation; abnormal activation in anterior and posterior instability has been found as well [15].

In summary, the combination of capsular laxity, altered scapular kinematics and muscle activity during elevation is believed to cause the glenoid to be positioned on a downward angle allowing the humeral head to be predisposed to escaping inferiorly [13]. As Heizelmann and Savoie [30] stated, *“the entire kinetic chain is disrupted, and the result is a cycle of inflammation leading to weakness, weakness leading to increased subluxation, and increased subluxation producing more inflammation. The inflammation leads to greater scapulothoracic malpositioning, causing both positional and functional weakness.”*

### 1.2 Clinical Presentation and Physical Examination

Clinical suspicion is essential in multidirectional instability, its diagnosis is complex to achieve and mainly clinical, needing a thorough patient history and systematic physical examination performed, as obvious as it is, with the patient undressed and examining both shoulders from the front as well as from the back.

Multidirectional instability typically produces pain and instability in the midrange positions of the glenohumeral motion, where a precise activation and control of the muscles is required, more so than end range stability, which depends to a greater extent on passive stabilizers.

The lack of consistent definition of multidirectional instability makes it difficult to clearly identify these patients, even more, it´s been demonstrated that variations in the definition of multidirectional instability significantly influence the number of patients who are included in the diagnosis [31].

First thing to have in mind in order to get to a correct diagnosis is that it´s critical to differentiate laxity from instability. Laxity in and of itself is not an indication for treatment and symptoms should be reproduced with examination maneuvers.

Usually these patients are in their second to third decade of life, and commonly their chief complaint will be a nonspecific pain, associated or not with other subtle symptoms covering a wide range from vague pain with no instability perception to frank instability. Sometimes they will refer a loss in their shoulder performance, either in daily activities that lead them to lifestyle changes in order to avoid certain positions and inciting activities, or in their strength and athletic performance.

Since the prevalence of multidirectional instability is higher in athletes involved in repetitive overhead sports, participation in such sports (as in throwing, volleyball, handball, swimming, gymnastics, weight trainers…), should be investigated.

Sometimes activity-related shoulder disability can help us to identify the direction of the instability, for example anterior instability when throwing or in overhead position, or discomfort carrying heavy objects that may be related to an inferior instability sometimes associated with numbness and paresthesias due to brachial plexus traction, or symptoms elicited by pushing (as in pushing open a door, push-ups or bench press exercises), that should alert us of posterior instability [12, 32, 33].

Other sources of pain around the shoulder have to be ruled out by examining the neck, acromioclavicular and sternoclavicular joints, even the elbow. Examination begins with inspection of the shoulder for previous scars, muscle atrophies, asymmetries, bone or joint deformities, and, more interestingly, the presence of scapular static malposition or winging, should be noted. General hyperlaxity should be assessed following either Bulbena´s Hospital del Mar Score or Beighton´s criteria. Bilateral shoulder range of motion and strength must be checked, and it may be normal but sometimes the patient will refer pain or apprehension.

In case we are suspecting instability and find generalized hyperlaxity criteria, we must ask for symptomatic laxity in other joints and if this is the case, determine if any connective tissue disorders exists.

Static observational test for scapular dyskinesis have failed to prove any difference between symptomatic and asymptomatic shoulders, although a careful observation of the resting and moving scapula will demonstrate scapular protraction in many multidirectional unstable patients, especially noted in the arm positions associated with instability symptoms. At this point, dynamic examination of the scapula, searching for signs of scapular dyskinesis, must be done. Since it has a great relationship with multidirectional instability it´s mandatory to be sure whether is present or absent.

The Scapular Dynamic Test (SDT) is a visual observation of the presence of either winging or dysrhythmia of the scapula while the patient performs repetitive weighted shoulder flexion and abduction.

**Fig. (2):**
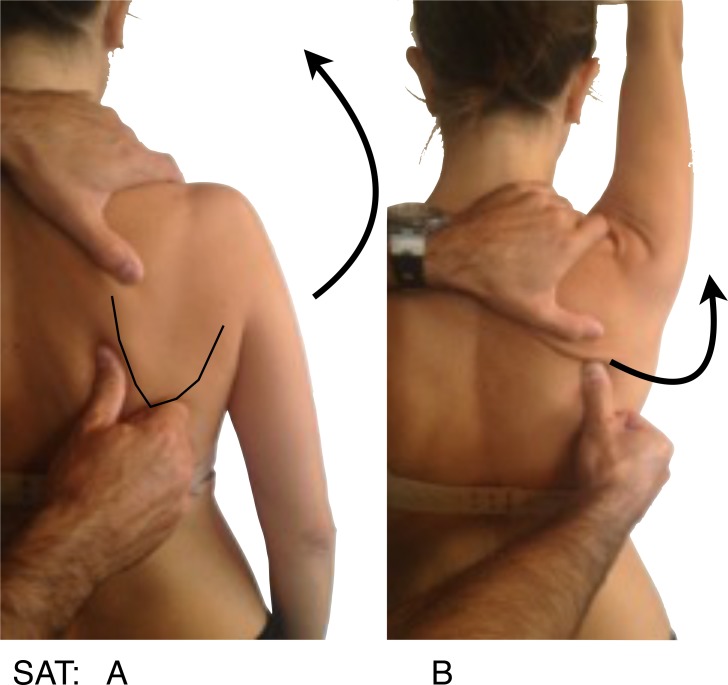
SAT: Scapular Activation Test, **A**: Thumb at the medial border of the scapula, the arrow indicates active forward elevation as seen in picture, **B**: Where arrow indicates how the thumb assists the upward elevation and posterior tilt.

Symptoms alteration test provide an excellent evidence that scapular dyskinesis is related to patient symptoms, if symptoms immediately decrease when we manually alter the scapular position during motion, these tests are:

The Scapular Activation Test (SAT) (Fig. **2**), where the examiner manually assists the upward rotation and posterior tilting during active shoulder elevation, and it´s considered positive when the pain is either decreased or abolished.

**Fig. (3):**
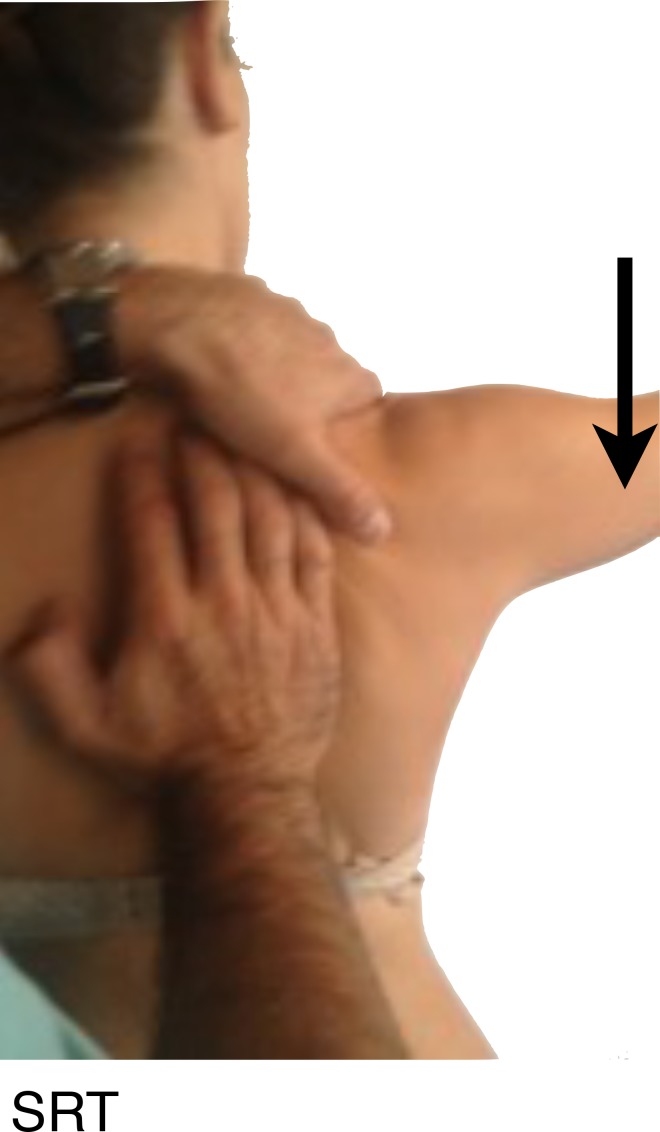
SRT: Scapular Retraction Test, Arrow indicates the resisted downward force that should be applied, before and after manual scapular stabilization as seen in the picture.

And the Scapular Retraction Test (SRT) (Fig. **3**), in which the examiner manually stabilizes and posteriorly tilts the medial border of the scapula, on a slight retracted position and test the elevation isometric strength in 90º on the scapular plane. It´s considered positive either if pain reduces or the strength increases. The SRT, by stabilizing the scapula in retraction, alters the glenoid position and decreases latissimus dorsi activation, and may decrease or eliminate the instability symptoms with arm motion [13].

During physical examination we should look for symptoms in the three most common directions, anterior, posterior and inferior. It should be noted once again that positive laxity tests cannot be considered as positive signs of instability. As well as a painful test, although positive, should be noted as painful test, not as instability. The most reliable result to consider instability is the provocation of apprehension with our examination tests.

**Fig. (4):**
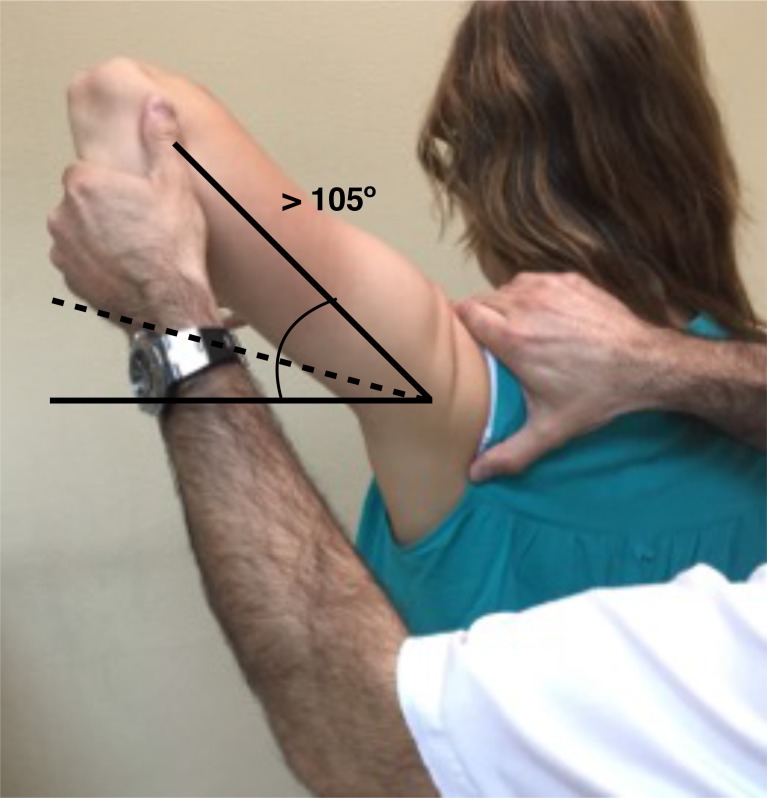
Gagey Test: Passive ABD greater than 105º.

In this context, signs for inferior laxity are performed not just to evaluate the laxity but to check if they reproduce the patient´s symptoms as well. The sulcus sign is positive if a subacromial sulcus appears when a caudal traction to the humerus is applied, performed in adduction and neutral rotation reflects an inferior laxity, that may be repeated in external rotation to test the rotator interval integrity and in in 90º of abduction to suggest participation of the inferior pouch to a capsular laxity. The hyperabduction test or the Gagey test (Fig. **4**) indicates inferior laxity when the passive abduction is greater than 105º or if an asymmetrical abduction with a difference greater than 20º is found.

**Fig. (5):**
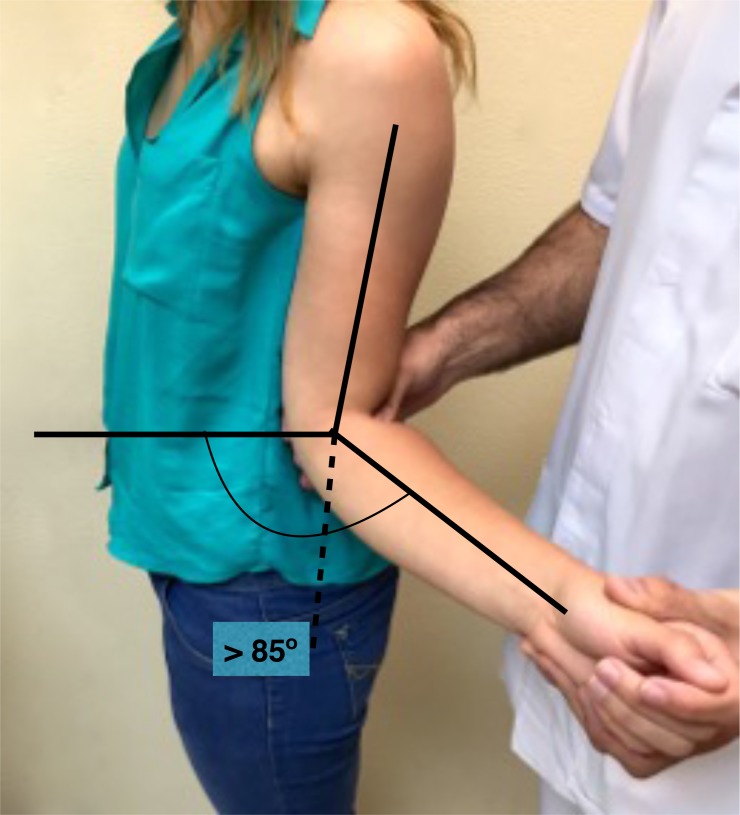
Hyper External Rotation: ER greater than 85º.

Anterior laxity can be confirmed when external rotation with the arm at the side is greater than 85º (Fig. **5**).

**Fig. (6):**
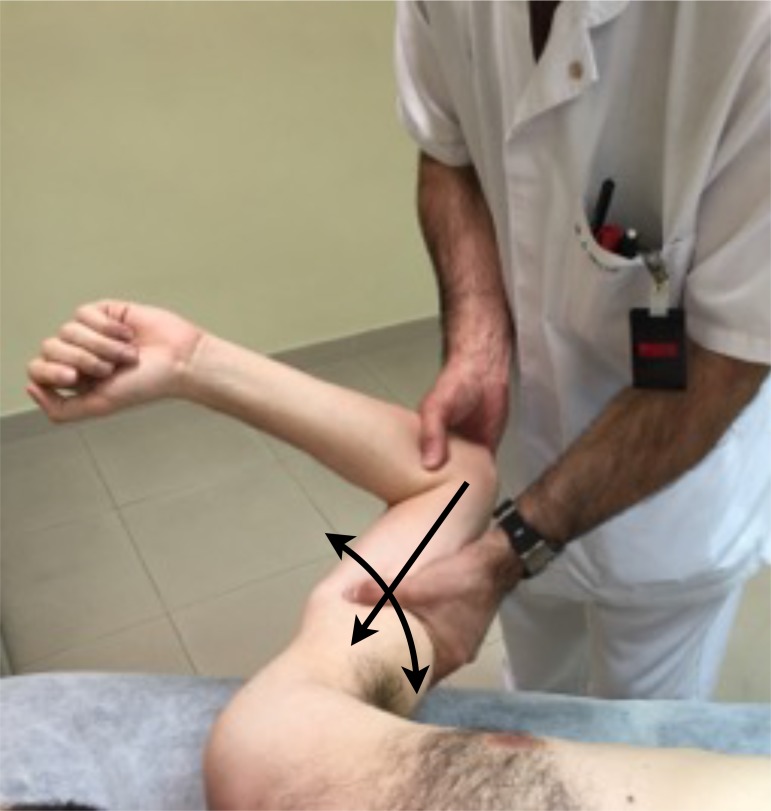
Load and Shift Test: Apply a small axial load then evaluate the translation of the humeral head anteriorly and posteriorly.

The **anterior and posterior load and shift** test (Fig. **6**), are a modification of the anterior and posterior drawer tests, and this test is performed with the patient supine and the arm in slight flexion and abduction, applying a small axial load, centering the humeral head into the glenoid, and then evaluate the translation of the humeral head anteriorly and posteriorly.

Sulcus, Gagey and load and shift tests are said to be the most sensitive in diagnosing a suspected multidirectional instability. Gagey has a well-defined cut-off point to be a positive test, but the other two are observational tests, so in order to be able to compare the results of these test we should use a grading system to quantify amount of translation and it´s been proposed a scale for translation of the humeral head that categorizes it as translation to the glenoid rim (grade I), translation over the glenoid rim, or dislocation with spontaneous reduction (grade II) and humeral head locked out or dislocation without spontaneous reduction (grade III). For the sulcus the established scale defines grade I as < 1.0 cm of sulcus, grade II as 1.0 to 2.0 cm and grade III as > 2 cm of displacement [31].

The provocation tests should also be performed, searching for signs of instability. The apprehension test, is performed passively placing the patient´s shoulder in the position of instability, abduction and external rotation for anterior instability or adduction and internal rotation for posterior instability, it is be considered positive if the patient feels apprehension in terms of involuntary guarding in the provocative position or senses pending subluxation. After this the relocation test is performed by applying a stabilizing counterforce in the provocative position alleviating the patient´s symptoms. We may confirm with a release test, where symptoms return when the stabilizing counterforce is released.

**Fig. (7):**
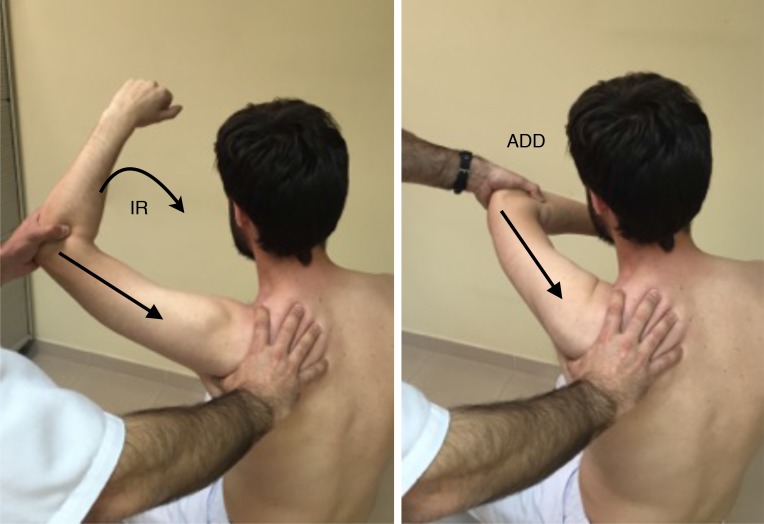
Jerk Test: Axial compression while internal rotation and horizontal adduction is applied.

**Fig. (8):**
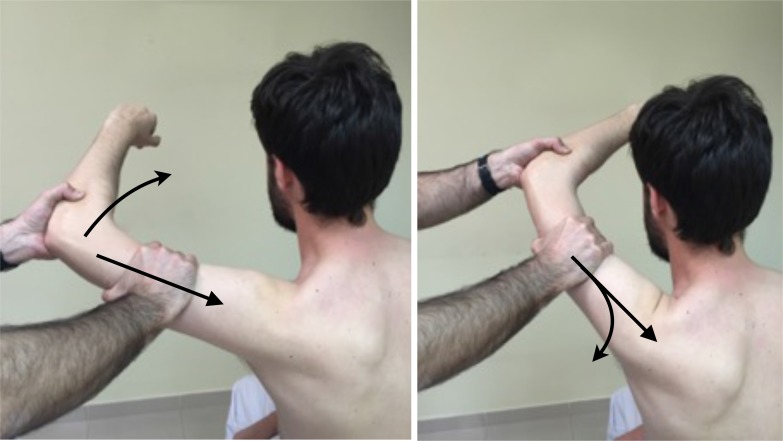
Kim Test: from abducted position a simultaneous axial load and a 45º oblique upward adduction is applied.

The posterior and posteroinferior labral lesions have two specific and sensitive tests. In the jerk test (Fig. **7**), the shoulder is placed at 90º of abduction and internal rotation, and while firmly producing an axial compression load throughout the test, the arm is moved horizontally to adduction across the body. A painful clunk is associated with a posteroinferior labral lesion. In the Kim test (Fig. **8**) the shoulder starts at 90º of abduction, and a simultaneous axial load and a 45º oblique upward adduction is applied, being positive if pain is elicited, regardless of associated posterior clunk. Kim test is more sensitive for predominant inferior labral lesion, while jerk test is more sensitive for predominant posterior labral lesion [34].

Examination under anesthesia should be performed when surgery has been decided. Since the evidence of augmented glenohumeral translation under anesthesia does not imply the existence of instability, we do not recommend this examination other than in the operating room as a preoperative preparation. In and of itself will not provide any diagnostic evidence, but combined with patient history, physical examination, may be of great help as diagnostic confirmation, more so in determining the real displacement in those patients that clinical examination was compromised by excessive pain and guarding.

**Fig. (9):**
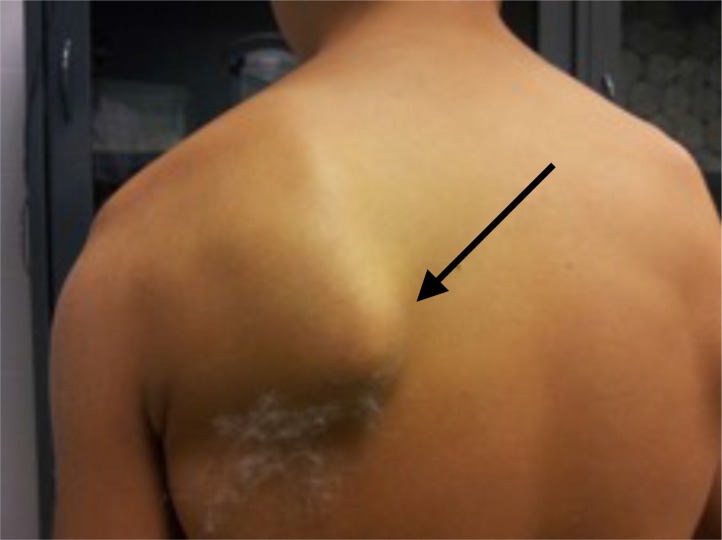
Typical scapular winging in voluntary dislocators.

Particular attention should be paid to the identification of voluntary dislocators, these patients, are commonly younger and use active voluntary muscle activation to subluxate, and tend to place the shoulder in internal rotation, with a typical winging of the scapula (Fig. **9**), either the medial inferior tip, similar to type I dyskinesia or the entire medial border as in type II dyskinesia. Also a sulcus sing and posterior subluxation when arm is actively placed in internal rotation are common findings [12].

Plain radiographs will not be of diagnostic value, but they should be performed and evaluated for glenohumeral alterations and bone deficiency, at the humeral head or glenoid rim. There´s no need for traction X-rays to prove inferior laxity.

In case we suspect bone deficiency a CT-scan in axial and coronal planes will clearly identify and quantify the bone defects. Nevertheless bony alterations are not common in multidirectional instability, so MRI remains the gold standard, better if intrarticular gadolinium contrast is used, since MR-arthrography allows for capsular distention and improves definition of the glenoid labrum and glenohumeral ligaments. Although labral abnormalities, and increased glenohumeral volume are often seen, these findings are nonspecific and may not reflect actual instability. Rotator interval augmentation or herniation has been pointed out as a key finding in multirectional instability [17], while other studies did not find any difference between different types of instability and control groups [35, 36].

**Fig. (10):**
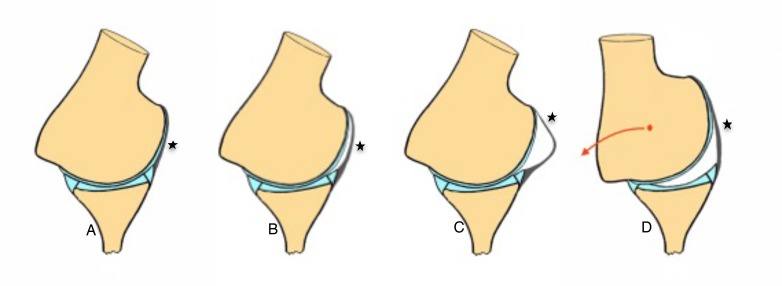
*AIGHL: Anteroinferior glenohumeral ligament. **A**: Normal image in ABER the AIGHL is tight against the humeral head, **B**: Crescent sign, **C**: Sigmoid, **D**: Triangle sign, due to posterior traslation of the humeral head.

New studies suggest that MR-Arthrogram performed in abduction and external rotation (ABER) offers better diagnostic value than regular MR-A. They describe two signs that combined allows for accurate and reproducible identification of patients with atraumatic multidirectional instability (Fig. **10**):

The crescent sign it’s a crescent shaped liquid between the anteroinferior glenohumeral ligament (AIGHL) and de humeral head either crescent or sigmoid shape are equally good for diagnosis since a normal shoulder would not show any liquid, due to the tighten anterior structures against the humeral head.

The triangle sign, just about the same sign but with a hyperabduction and decentering of the humeral head displaying that triangle shape between the humeral head, articular surface of the glenoid and AIGHL [36]. The combination of the two signs have shown a sensitivity of up to 90% and a specificity of 94%.

In the case we want to take a step forward and assess abnormal muscle activation patterns, dynamic electromyography (DEMG) offers useful diagnosis, since nearly in half of the clinically suspected abnormal muscle patterning, the specific muscles are incorrectly identified. It also may be of importance in order to characterize specific pathology and develop specific rehabilitation protocols for each patient.

### 1.3 Natural History

Natural history of this condition has not been clearly studied in the reviewed literature, in fact the search on databases for this topic does not yield any result. We should be aware that the wide variation of multidirectional instability patterns and patients, all together with the multifactorial nature of multidirectional instability and the variety of definitions within the available literature, makes very complex to compare different studies, and many draw opposite conclusions.

We may assume that as a chronic instability that it is, the evolution to articular degeneration and arthropathy is warranted, but the fact is that bone and chondral damage are not as common as in traumatic recurrent instability.

Since a global dysfunction of the shoulder occurs, scapular malposition and dyskinesis may cause other related alterations and cuff problems may be expected in these patients in the long term.

Quality of life in multidirectional unstable patient will probably be low and lowering with an increasing apprehension if subluxations are not controlled and damage to capsulolabral structures increases. The only study found about this topic is the one of Merolla *et al*. [37], where they studied the influence of making the patients aware of abnormal muscle patterns, teach them how to restore scapular motion and encourage the adoption of home rehabilitation exercises as a part of their normal lifestyle, in a voluntary posteroinferior unstable group. They found that at the end of the study most of the patients were still able to subluxate their shoulders, but were satisfied and had increasing values in the scores applied during follow-up. Also, stratifying outcomes by age, found that the higher values were in patients aged 12-18 years, therefore hypothesized that early treatment might ensure better pain and quality-of-life outcomes. The fact that patients who did not engage in rehabilitation of the scapulo-humeral rhythm and muscle balance had the lowest outcomes and needed surgery due to persistent pain and instability; together with the fact that at the end of the study in spite of good results, the patients were still voluntary dislocators, but had the situation under control, leads us to assume that the natural history of the multidirectional unstable patient will be highly related to their ability to control scapulo-humeral kinesis.

## CONCLUSION

The diagnosis of a patient as multidirectional unstable should be done according to clinical evidence of symptoms in two or more directions, not by laxity tests, since it´s known that the passive subluxation of the humeral head over the glenoid rim should only be considered instability if it produces symptoms [31].

Many differences can be seen in the current literature when identifying these patients, unclear definitions and criteria to be included in this patient group are common. But in general, we can make a very close picture of what is going on with our patients, and the importance of the global assessment of the shoulder function has to be highlighted. There is need for a whole new generation of data based on clear inclusion criteria, classification and subdivisions of multidirectional instability (patients with posteroinferior instability are probably not going to have the same anatomic alterations as seen in anteroinferior unstable patients), so we can study neuromuscular control alterations, anatomic alterations and clinical findings in every group of multidirectional unstable patients in order to identify the best treatment for our patients.

## Figures and Tables

**Table 1:** Modified Beighton´s Criteria for Hiperlaxity.

Assessment site	Right	Left
Hyperextension of elbow > 10º	1	1
Thumb touching forearm	1	1
Hyperextension of 5th metacarpal joint > 90º	1	1
Hyperextension of knee joint > 10º	1	1
Forward flexion of trunk, palms rest flat on the floor with knees fully extended	1	
Maximum possible Score	9	

Hypermobility present if score equals or higher than 4.

**Table 2:** Hospital del Mar Score (10 items).

Assessment site		Yes	No
Upper extremity	Thumb: passive apposition of the thumb to the flexor aspect of the forearm at less than 21mm	1	0
	Metacarpophalangeal: passive dorsiflexion of 5th finger of 90º or more	1	0
	Elbow hyperextension: passive extension of 10º or more	1	0
	External shoulder rotation: elbow flexed and upper arm touching the body, a passive rotation of 85º or more.	1	0
Lower extremity Supine position	Hip abduction: passive of 85º or more	1	0
	Patellar hypermobility: defined as excessive passive displacement medially and laterally as assessed by three or more quadrants of displacement	1	0
	Ankle and Feet: Excessive range of passive ankle dorsiflexion and eversion of the foot with the knee flexed to 90º	1	0
	Metatarsophalangeal joint: hyperextension of the first toe beyond 90º	1	0
Lower extremity Prone position	Knee hyperflexion: defined as “passively the knee makes contact with the buttock”	1	0
	Ecchymoses: appearance of ecchymoses after hardly noticed, minimal trauma (historical datum)	1	0

Score of 4 or more for men, and 5 or more for women, suggests the presence of generalized joint laxity.

**Table 3:** Gerber and Nyfeller´s classifcation of dynamic shoulder instability (B).

**Classification**	**Description**
B1: Chronic Locked Dislocation	Locked instability caused by major trauma
B2: Unidirectional Instability without hyperlaxity	Symptoms elicited in a single direction Traumatic capsulolabral lesions frequently present
B3: Unidirectional instability with hyperlaxity	Symptoms elicited in a single direction Patulous capsular tissue frequently present Presence of capsulolabral lesion less likely
B4: Multidirectional Instability without hyperlaxity	Symptoms elicited in two or more directions Anterior and posterior capsulolabral lesions frequently present
B5: Multidirectional Instability with hyperlaxity	Symptoms elicited in two or more directions Patulous capsular tissue frequently present Signs of generalized hyperlaxity frequently present Frequent recurrent subluxation
B6: Uni or Multidirectional with voluntary reduction.	At first dislocation is not noticed and voluntary reduction is symptomatic. With time they learn to put the shoulder in dislocation position and reduce it.

## LIST OF ABBREVIATIONS

ABER: = Abduction and External Rotation
AIGHL: = Anteroinferior Glenohumeral Ligament
AMBRI: = Atraumatic, Multidirectional, Bilateral, Rehabilitation, Inferior capsular shift
CT-scan: = Computed Tomography scan
DEMG: = Dynamic Electromyography
MDI: = Multidirectional instability
MR-arthrography and MR-A: = Magnetic Resonance Arthrography
MRI: = Magnetic Resonance Imaging
SAT: = Scapular Activation Test
SDT: = Scapular Dynamic Test
SRT: = Scapular Retraction Test
TUBS: = Traumatic, Unidirectional, Bankart, Surgery
